# Preliminary Results on the Added Value of Parametric Images Derived from ^18^F-fluoroethyl-L-tryptophan PET for Posttreatment Glioblastoma Assessment

**DOI:** 10.1007/s11307-025-02075-4

**Published:** 2025-12-22

**Authors:** Csaba Juhász, Geoffrey R. Barger, Michael Dominello, Natasha L. Robinette, Parthasarathi Chamiraju, Huailei Jiang, Otto Muzik

**Affiliations:** 1 Department of Pediatrics and Translational Imaging Laboratory, Wayne State University School of Medicine, Detroit, MI USA; 2 Department of Neurology, Wayne State University School of Medicine, Detroit, MI USA; 3 PET Center, Barbara Ann Karmanos Cancer Institute, Detroit, MI USA; 4 Department of Radiation Oncology, Barbara Ann Karmanos Cancer Institute, Wayne State University School of Medicine, Detroit, MI USA; 5 Department of Radiology, Wayne State University School of Medicine, Detroit, MI USA; 6 Department of Neurosurgery Detroit Medical Center, Detroit, MI USA; 7 University Health Center, Suite 4J-B, 4201 St. Antoine, Detroit, MI 48201 USA

**Keywords:** Positron emission tomography, Tryptophan metabolism, [^18^F]-fluoro-ethyl-L-tryptophan, Kynurenine pathway, Glioblastoma, Post-treatment

## Abstract

**Background:**

Increased amino acid transport in gliomas allows imaging of metabolically active tumor volume by PET. Tryptophan analog PET radiotracers can provide additional information by tracking tumoral metabolism via the upregulated immunosuppressive tryptophan-kynurenine pathway. We tested the recently developed tryptophan analog PET tracer [^18^F]-fluoro-ethyl-L-tryptophan ([^18^F]FETrp) for detecting post-treatment glioblastoma while using a non-invasive approach to generate parametric tryptophan metabolic maps and comparing them with static tracer uptake maps and contrast-enhanced MRI.

**Methods:**

Five patients (age: 22–67 years) with previously treated glioblastoma underwent [^18^F]FETrp PET/CT imaging. A dynamic acquisition protocol sampled the brain and blood pool non-invasively using the FlowMotion Multiparametric PET software (Siemens Healthineers). Parametric brain images of the unidirectional uptake rate constant (K_i_), characterizing irreversible tryptophan trapping and the volume of distribution (V_D_) were fused with static uptake (SUV) maps and contrast-enhanced brain MRI. Voxels with elevated K_i_, V_D_, and SUV were defined, and their spatial associations with contrast-enhanced volumes were characterized by their % volume overlap and the distance between their centroids.

**Results:**

A substantial spatial volume overlap was observed between MRI contrast-enhancing regions and elevated static [^18^F]FETrp uptake and V_D_. In contrast, the overlap between contrast-enhancing regions and elevated K_i_ metabolic volumes was low (0–16%), with high K_i_ areas extending deeper into non-enhancing brain (7–33 mm centroid distance). These non-enhancing high K_i_ areas showed new contrast-enhancement on follow-up MRI, consistent with tumor progression.

**Conclusions:**

Areas of high tryptophan metabolism detected by [^18^F]FETrp PET-derived parametric (K_i_) maps extend outside the contrast-enhancing glioblastoma mass in adjacent non-enhancing brain regions that can be missed or underestimated by static uptake images. Consequently, [^18^F]FETrp PET metabolic maps have the potential for enhanced detection of non-enhancing glioma infiltration for improved radiation or surgical treatment planning.

**Supplementary Information:**

The online version contains supplementary material available at 10.1007/s11307-025-02075-4.

## Introduction

Brain tumors remain a major health issue with dismal outcomes. Malignant brain tumors remain among the deadliest cancers, especially in patients with WHO grade IV glioma (glioblastoma) who suffer the worst outcome (median survival of 15 months) [1]. At present, anatomical magnetic resonance imaging (MRI) remains the main modality for accurate diagnosis, treatment planning and response assessment in patients with gliomas [2–4]. Unfortunately, the radiographic features and findings of glioma, even utilizing advanced MRI sequences such as spectroscopy, diffusion and perfusion imaging, are often ambiguous, particularly regarding the delineation of non-enhancing tumor infiltration and differentiation of treatment-induced changes from actual tumor progression [5]. This is partly due to the limitation of standardized anatomical brain tumor MRI protocols to adequately assess metabolic tumor activity [6]. While positron emission tomography (PET) imaging holds promise to overcome these limitations, the widely used [^1^⁸F]FDG is suboptimal for gliomas partly due to high physiological uptake in normal cortex and basal ganglia and its limited specificity to differentiate malignant glioma progression from treatment-induced changes after radiation therapy [7, 8].

Over the past decade, amino acid PET tracers have gained prominence in brain tumor imaging due to their ability to provide information about the transport and, to a limited extent, metabolism of specific amino acid molecules. Various amino acid PET tracers, including [^11^C]methionine, [^18^F]fluoroethyl-tyrosine and [^18^F]fluoro-DOPA, enable the evaluation of amino acid transport from the bloodstream into tumor tissue. Based on accumulating data, amino acid PET has been endorsed by various professional organizations for: (i) diagnosing/grading brain tumors, (ii) delineating extent for resection or radiation planning, (iii) monitoring treatment, and/or (iv) diagnosing post-treatment progression and differentiating them from treatment-induced changes [9–12].

Despite their clinical utility in quantifying amino acid transport into tumors, currently available amino acid PET tracers exhibit several limitations that constrain their ability to fully characterize tumor biology. A primary limitation is that the predominant mechanism of tumor uptake relies on transport via the L-type amino acid transporter system, with minimal contribution from amino acid metabolism, thus restricting the biological specificity of the PET signal. Furthermore, existing amino acid tracers lack a direct mechanistic association with established pathways of tumor-associated immunosuppression, limiting their potential in assessing the tumor microenvironment. Another shortcoming is the predominant reliance on static PET imaging in clinical applications, which fails to exploit the full potential of dynamic imaging and tracer kinetic modeling. Although recent studies have employed semiquantitative parametric analysis derived from dynamic PET imaging, demonstrating potential in glioma grading and the prediction of isocitrate dehydrogenase (IDH) mutation status [13, 14], these methods do not enable the direct quantification of tracer transport kinetics or metabolic trapping in the absence of tumor-specific metabolic interactions. Overcoming these limitations by generating parametric images based on tracer kinetics and developing novel metabolically active tracers could therefore significantly enhance the diagnostic and prognostic capabilities of amino acid PET imaging in neuro-oncology.

In our previous studies employing alpha-[^11^C]methyl-L-tryptophan ([^11^C]AMT), we implemented kinetic modeling using dynamic PET imaging combined with an image-derived blood input function to quantitatively assess both tryptophan transport and tryptophan metabolism in brain tumors [15–18]. This approach allowed the characterization of metabolic changes associated with both serotonin synthesis and tumor-associated immunosuppressive mechanisms, specifically those mediated by the kynurenine (KYN) pathway, which plays a critical role in the tumor microenvironment and can contribute to tumor progression and poor survival associated with malignant gliomas [19–26]. However, the short half-life of carbon-11 (20 min) and cumbersome radiosynthesis of [^11^C]AMT imposed significant constraints on the clinical applicability of [^11^C]AMT PET. To address these limitations, we focused on the development of an [^18^F]-labeled tryptophan derivative with enhanced suitability for evaluating tumoral tryptophan metabolism. Among over a dozen [^18^F]-labeled tryptophan analogs investigated in preclinical studies [27], 1-(2-[^18^F]fluoroethyl)-L-tryptophan ([^18^F]FETrp) emerged as the most promising candidate for clinical translation. The uptake and metabolic trapping of [^18^F]FETrp reflect not only amino acid transport but is sensitive to the activity of indoleamine 2,3-dioxygenase 1 (IDO1), an enzyme that represents the rate-limiting step in the KYN pathway, as confirmed by preclinical studies [28, 29]. As a consequence, the metabolic rate constant derived from kinetic analysis allows the assessment of tryptophan metabolism via KYN pathway activity under pathological conditions.

Moreover, recent advances in PET scanner technology and software have introduced the possibility of dynamic multi-pass PET/CT data acquisition [30]. This technique allows the continuous scanning of multiple whole-body passes covering both the brain and the heart and the extraction of an arterial image-derived input function (IDIF). This data is then used for direct reconstruction of multiparametric images based on the linear Patlak analysis [31, 32]. Such multiparametric imaging complements the standard SUV image with two new parametric images: one representing the net influx rate constant of the tracer into tissue (K_i_) and another representing the apparent distribution volume (VD) of the tracer in the reversible tissue compartment.

Multiparameter imaging has proven to be feasible in a clinical setting [33] and demonstrated improved tumor lesion detectability in whole-body FDG images with reduced false-positive rates when complementing standard-of-care SUV imaging [34]. However, this technique has so far not been applied to other tracers than FDG and has not been utilized in the diagnostic assessment of brain tumors. Our recent study introduced the use of [^18^F]FETrp PET in detecting human gliomas, including non-contrast enhancing tumor portions [35]. In the present study, we investigated the utility of dynamic [^18^F]FETrp PET imaging, in conjunction with multiparametric imaging and the generation of parametric images representing irreversible tracer uptake, for the delineation of tumor tissue extent in patients with glioblastoma. This approach included the characterization of non-enhancing tumor-infiltrated brain regions detected by [^18^F]FETrp PET, whose precise pre-treatment identification may facilitate improved targeted interventions in tumors showing post-treatment progression.

## Methods

### Subjects

Five patients (all males; age, 22–67 years; see Table 1) participated in this study. All patients had been previously treated with glioblastoma, including surgical resection with (*n* = 4) or without (*n* = 1) subsequent post-surgical chemoradiation, and follow-up clinical imaging showed contrast-enhancing lesion(s) suspicious for post-treatment tumor persistence or progression (Table 1). This included a progressive MRI contrast-enhancing lesion in four patients and brain CT showing a peri-resection abnormality suspicious for persistent tumor after a recent re-resection (patient #4). All [^18^F]FETrp PET/CT scans were acquired within a 2-week window following these clinical imaging acquisitions, but at least 4 weeks after a previous intervention (surgery or chemoradiation). Tumor progression was verified after the [^18^F]FETrp PET/CT scan by histopathology or follow-up MRI showing progressive changes in all five patients (Table 1). Additional inclusion criteria were as follows: (i) age ≥ 18 years; (ii) targeted lesion (presumed tumor) is at least 1 cm in diameter as shown by clinical imaging; (iii) no tumor surgery or radiation treatment within 1 month. The study was approved by the Wayne State University Institutional Review Board, and all participants signed a consent form.

**Table 1:** Patients’ age and tumor data. IDH status refers to the original histopathology. Post-treatment tumor location refers to region(s) suspicious for post-treatment tumor progression/persistence on clinical imaging. These were then verified by histopathology (from re-resection) or MRI(s) performed after the PET scan

Pt #	Age (y)	IDH status	RT	Progr. tumor location	Post-PET tumor verification
1	67	wild type	No	Left temporal, parietal	Histopathology
2	63	mutated	Yes	Right frontal	Progressive MRI enhancement
3	63	wild type	Yes	Left parietal	Progressive MRI enhancement (5 mo)
4	22	wild type	Yes	Right frontal-insular	Histopathology
5	43	wild type	Yes	Right frontal	MRI enhancement

*RT* history of radiotherapy

### Radiotracer Synthesis

[^18^F]FETrp was synthesized following specifications described in FDA IND #162,122 using a Synthra RNplus module as described recently [36]. [^18^F]FETrp was obtained with high (> 99%) radiochemical purity and had specific radioactivity of 226 ± 19 GBq/μmol at the time of injection. The mean and standard deviation of the administered mass of [^18^F]FETrp were 0.78 ± 0.40 μg (range, 0.35–1.50 μg). The mean administered activity was 470 ± 140 MBq (300–630 MBq). High-performance liquid chromatography showed tracer stability in mouse serum at 37 °C after 2 h, with > 95% of the radiotracer remaining intact [28]. Although no similar human data are available, the favorable metabolic profile in rodents supports the use of an image-derived input function in PET studies without metabolite correction.

### PET/CT Acquisition

All scans were performed using a Siemens Vision 600 PET/CT scanner (Siemens Healthineers, Knoxville, USA) with 213 ps time-of-flight capability and supporting continuous bed motion (CBM), which allows dynamic multi-pass image acquisition at multiple levels of the body (e.g., the brain and the heart). Subjects were fasted for at least 6 h prior to the PET imaging session to ensure stable blood tryptophan levels. A venous line was established for tracer injection (5.2 MBq/kg). Prior to tracer injection a low-level CT scan (100 keV, 80 mA) was acquired for attenuation correction. The tracer was injected as a slow bolus over 30 s using a Bayer Medrad Stellant CT injector system. Coinciding with tracer injection, a dynamic image sequence (12 × 10 s, 6 × 30 s; total 5 min) at the level of the heart was initiated to obtain an accurate representation of the bolus part of the left-ventricular (LV) arterial input function. During the rest of the PET imaging protocol, the scanner used CBM to move the patient’s head and upper torso in and out of the field-of-view, so that dynamic emission data from both the brain and the heart could be collected. This multi-pass protocol (11 × 5 min) yielded dynamic brain and heart data from 5–60 min post-injection (p.i.). All PET images were corrected for attenuation, tracer decay and scatter and reconstructed using the OSEM iterative reconstruction algorithm yielding dynamic brain and heart images with isotropic voxel size (1.65 mm^3^). The vital signs of all subjects were recorded before radiotracer injection and again at the conclusion of the PET scan.

### Parametric Image Generation

The automated multiparametric scan protocol automatically defined an LV region of interest (ROI) within the CT image volume and subsequently transferred this ROI to both the initial dynamic PET scan of the heart and the multi-pass CBM scan at the cardiac level. Time-activity curves (TACs) representing LV activity were then extracted from both imaging sequences and concatenated to construct a complete image-derived input function (IDIF) spanning 0–60 min p.i. Plots of representative IDIFs extracted from the data are provided in the Supplementary Data section. Validation studies [37] have demonstrated that image-derived input functions obtained from the descending aorta or left ventricular regions enable generation of Patlak-derived K_i_ maps that closely approximate those obtained with arterial blood sampling (SSIM > 0.98, r^2^ > 0.88). Parametric images were directly reconstructed from raw data using the FlowMotion Multiparametric PET software from Siemens Healthineers. Specifically, a voxel-wise Patlak graphical analysis was performed using the IDIF and the multi-pass CBM image sequence acquired at the level of the brain. This analysis yielded parametric images representing the slope and intercept of a linear function fitted to the Patlak-transformed IDIF and voxel-wise tissue TACs from 35–60 min p.i. The slope parameter corresponds to the unidirectional uptake rate constant K_i_, which characterizes the irreversible trapping of the tracer in tissue. The intercept parameter represents the apparent volume of distribution (V_D_), reflecting the initial tracer distribution within the tissue before irreversible binding occurs. The V_D_ parameter reflects a combination of vascular volume, non-trapped tracer and reversible binding.

The use of Patlak linearization was motivated by prior work with the closely related tryptophan analog [^11^C]AMT, for which we have previously established a two-tissue irreversible compartment model describing tracer transport, reversible exchange, and metabolic trapping via serotonin and kynurenine pathways [38]. This prior validation demonstrated that Patlak analysis provides an accurate linear approximation of net metabolic trapping for tryptophan derivatives. Consistent with these findings, preclinical investigations of [^18^F]FETrp have confirmed similar kinetics, including blood–brain barrier penetration, metabolic conversion via IDO, and irreversible trapping in tumor tissue [29, 39]. Based on this biochemical and kinetic evidence, Patlak analysis was selected as a robust and biologically appropriate quantification method for [^18^F]FETrp. To demonstrate the appropriateness of this approach, sample time-activity curves fitted with a two-tissue compartmental model as well as the corresponding Patlak-plot graphs are provided in the Supplementary Data section. Nevertheless, future studies are warranted that apply full compartmental modelling in combination with a metabolite corrected, blood sampled input function in order to validate the use of an IDIF in conjunction with the Patlak analysis for the [^18^F]FETrp tracer in a larger cohort study.

### MRI Acquisition

The MRI scans were performed clinically using a Philips Ingenia Elition 3.0 Tesla, 70 cm short bore, 55 cm field-of-view clinical MR scanner. The brain tumor protocol includes 4 mm sagittal T1 TSE, 3 mm axial T1 SE, T2 TSE SPAIR, fluid-attenuated inversion recovery (FLAIR), diffusion-weighted imaging, venous BOLD (3D GRE), post-contrast axial T1 SE, 1.0 mm sagittal 3D FLAIR with axial and coronal reformatted images, and post-contrast 1.0 mm sagittal T1 3D TFE with coronal and axial reformatted images. Advanced sequences routinely included single voxel, 2D or 3D multivoxel PRESS MR spectroscopy and MR T2* first pass dynamic perfusion imaging with injection flow rate of 1.5 mL/s (0.1 mL/kg) gadobutrol (Gd) as a paramagnetic macrocyclic contrast agent.

### Multi-Modality Analysis

Initially, a summed PET image was generated representing the overall tracer distribution within the brain. Subsequently, structural MRI sequences including native T1-weighted (T1-w), gadolinium-enhanced T1-weighted (T1-Gd), and FLAIR images were co-registered to the summed PET image. In addition, standardized uptake value (SUV) images were generated by averaging the tracer distribution in the brain over the last 2 frames of the dynamic sequence (50–60 min p.i.), when tumoral uptake plateaus [35]. This approach enabled a spatial correlation analysis between the various MRI sequences and SUV, Ki, and VD parametric images.

Image analysis was performed using the PMOD 4.5 software (PMOD Technologies, Zurich, Switzerland). Gadolinium-enhancing regions on T1-Gd MR images were identified using a semi-automated intensity thresholding method. Specifically, a difference image was generated by subtracting the (co-registered) pre-contrast from the post-contrast T1-weighted image, highlighting voxels with signal enhancement attributable to gadolinium uptake in the presumed tumor region. To quantify the spatial relationship between the so defined contrast-enhancing regions observed on MRI and metabolic activity of tryptophan measured via PET imaging (SUV, K_i_, and V_D_ maps), normative control values and their corresponding standard deviations (SDs) were first established by delineating homotopic regions within the contralateral (presumed normal) hemisphere in cortex with normal MRI appearance. Abnormal regions within the ipsilateral hemisphere were subsequently identified by applying a threshold of greater than 2 SDs above the mean control values. The spatial association between these abnormal regions was then characterized using two metrics: (i) the percentage overlap relative to non-overlapping areas, and (ii) the Euclidean distance between the centroids (centers of gravity) of the respective regions.

### Statistical Analysis

Two-sided paired t-tests were employed to compare regions with abnormally elevated SUV, K_i_, and V_D_ values, as well as structural abnormalities defined by T1-gadolinium enhancement, within each subject's brain. Additional paired t-tests assessed the significance of percentage overlaps and centroid distances among these regions. Statistical significance was set at *p* < 0.05.

## Results

There were no adverse or clinically detectable pharmacologic effects in any subjects, and no significant changes in vital signs as a result of the imaging procedure.

### Normative Values for K_i_, V_D_ and SUV

Control cortical values for K_i_ derived from the contralateral hemisphere were 0.00308 ± 0.00026 (Coefficient of Variation [COV] = 8.4%), whereas cortical control values for V_D_ and SUV were found to be 0.26 ± 0.03 (COV = 11.5%) and 0.67 ± 0.10 (COV = 15%), respectively. Consequently, values greater than 2SD above these normative values were considered as abnormal in the ipsilateral hemisphere (i.e., values greater than 0.00360 for K_i_, 0.32 for V_D_ and 0.87 for SUV). Representative time-activity curves derived from control cortical areas are provided in the Supplementary Data section.

### Tumor-Associated K_i_, V_D_, and SUV Values

**Fig. 1:**
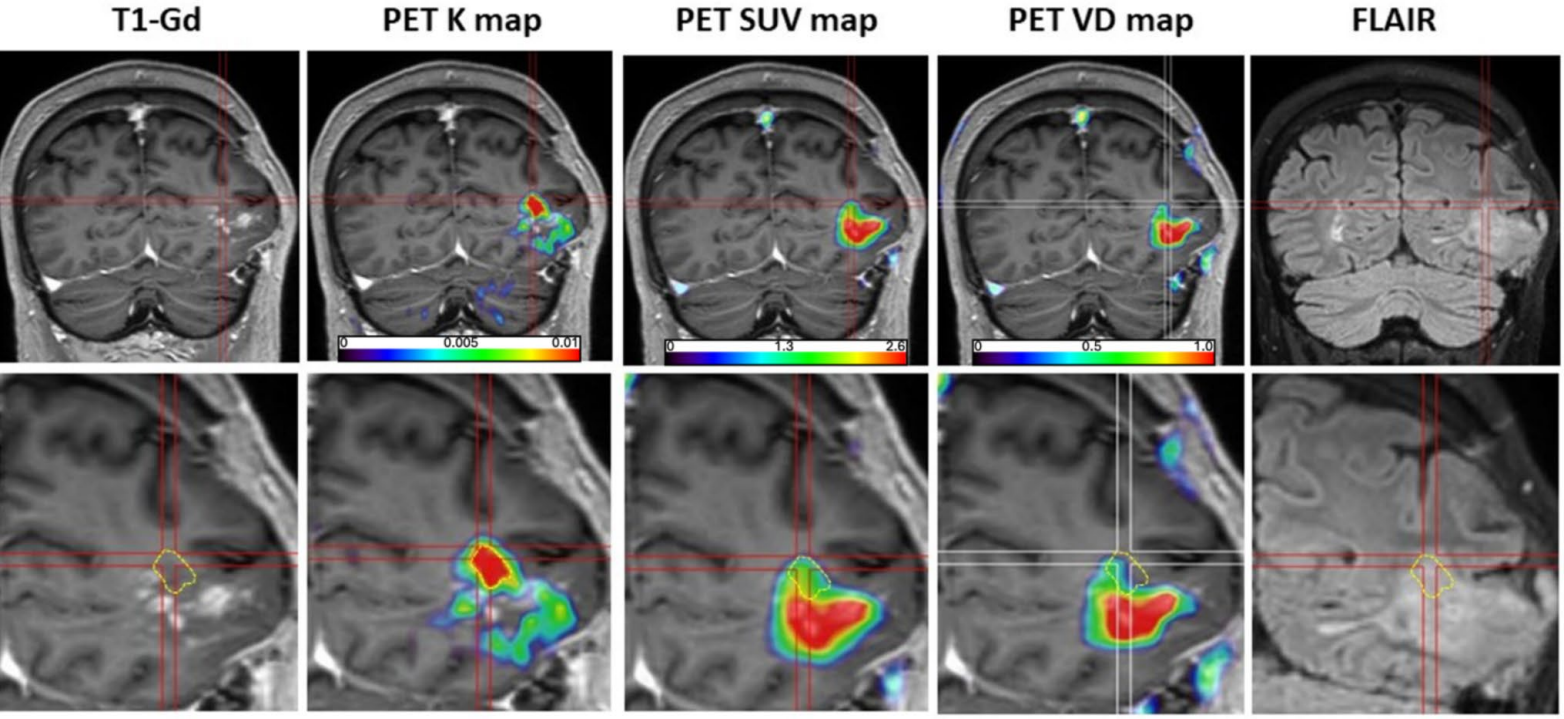
Representative co-registered multimodal images from a patient (#1) with an area of heterogeneous contrast enhancement in the left parietal lobe, suspicious for post-treatment tumor progression. Coronal post-gadolinium T1 MRI (T1-Gd) image shows the contrast-enhancing area, which is then fused with [^1^⁸F]FETrp PET images, including the parametric K_i_ map, static SUV map, and V_D_ map, where the red areas represent voxels with high values (> 2 SD from contralateral normal cortex). The bottom panels show the same area enlarged. The regions of elevated SUV and V_D_ largely colocalize with gadolinium enhancement, consistent with blood–brain barrier disruption. In contrast, the area demonstrating increased K_i_ values—indicative of enhanced unidirectional tracer influx and metabolic conversion and outlined with yellow dotted lines – is anatomically distinct and is localized largely in adjacent, non-enhancing brain, encompassing a portion of the FLAIR abnormality, suggesting tumor infiltration into non-enhancing brain beyond areas of blood–brain barrier compromise. Histopathology from re-operation showed massive tumor infiltration in this area. Corresponding time-activity curves of this patient representing regions with high K_i_ and high SUV values together with Patlak-plots are provided in the Supplementary Data section

Regions displaying elevated K_i_ values indicative of increased [^18^F]FETrp trapping due to active tumor tissue were identified from parametric K_i_ images, with an average K_i_ value of 0.0112 ± 0.0029, significantly higher compared to control cortical regions (*p* = 0.028). Corresponding V_D_ and SUV values in these high K_i_ regions were 0.39 ± 0.16 and 2.25 ± 0.82, respectively. Figure 1 illustrates a representative example, showing regions with high K_i_ values distinctly separated from regions of elevated tracer uptake (SUV), typically localized at the periphery and extending beyond contrast-enhancing mass. Additionally, representative time-activity curves derived from tumor tissue characterized by high SUV or elevated K_i_ values are provided in the Supplementary Data section.

**Fig. 2:**
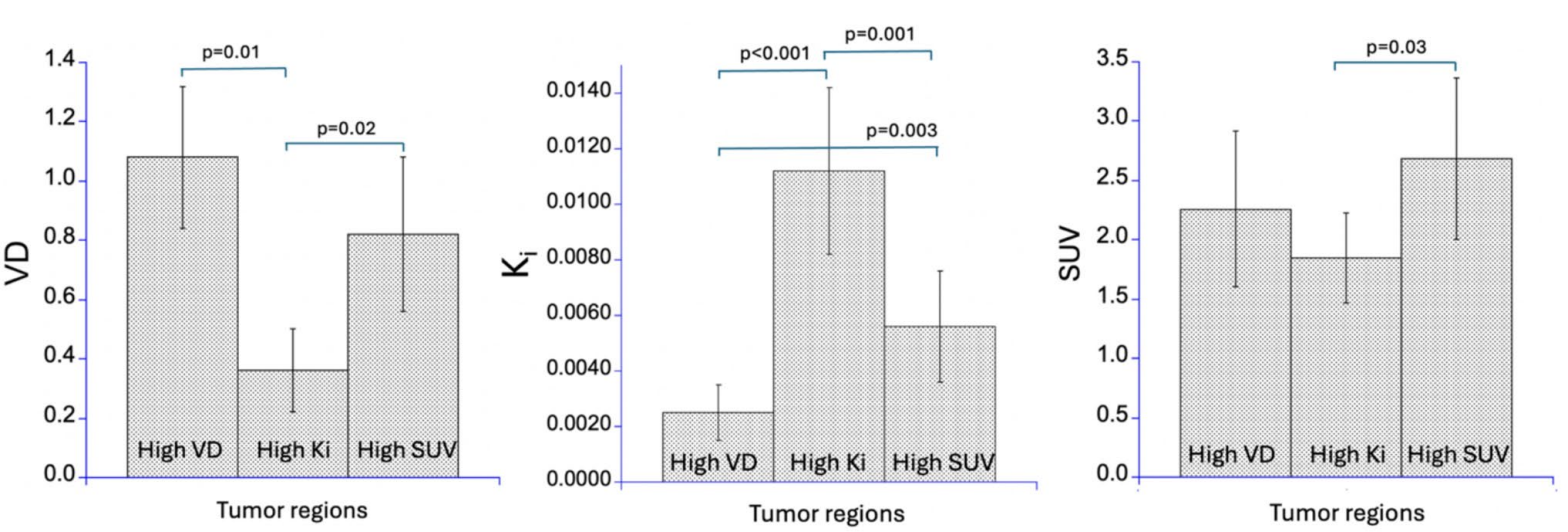
Graphical comparison of volume of distribution (V_D_; left), irreversible uptake constant (K_i_; middle), and standardized uptake value (SUV; right) across regions of interest (ROIs) defined by elevated V_D_, K_i_, and SUV, respectively. K_i_ values were significantly higher in ROIs defined by elevated K_i_ compared to those defined by elevated V_D_ or SUV, indicating that metabolic trapping of [^1^⁸F]FETrp is distinct from nonspecific tracer accumulation. A strong concordance between SUV and V_D_ values was observed within their respective ROIs, consistent with passive tracer distribution (due to leakage through the impaired blood-tumor-barrier) and/or increased amino acid transporter activity. In contrast, ROIs with high K_i_ showed comparatively lower V_D_ and SUV, reflecting predominant metabolic trapping rather than nonspecific uptake, highlighting the functional distinction between these PET measures. Statistical significance was assessed using two-sided paired t-tests

Table 2 shows individual V_D_, SUV, and K_i_ values in regions identified by abnormally elevated V_D_, SUV and K_i_ values. K_i_ values in regions with abnormally elevated K_i_ values were about twice as large as in areas with abnormally elevated SUVs (0.0112 ± 0.0029 vs 0.0056 ± 0.0020, *p* = 0.001) and about four times as large as in areas with abnormally elevated V_D_ values (0.0112 ± 0.0029 vs 0.0025 ± 0.0010, *p* < 0.001). Moreover, regions identified by elevated V_D_ showed significantly higher V_D_ values (1.09 ± 0.24) compared to regions identified by high K_i_ (0.34 ± 0.15; *p* = 0.01), while remaining similar to regions with elevated SUV (0.83 ± 0.27). The SUVs were found to be highest in regions identified by elevated SUV values (2.63 ± 0.58) and similarly elevated in regions with high V_D_ (2.18 ± 0.97), but they were significantly lower in regions characterized by high K_i_ (1.85 ± 0.44, *p* = 0.03). Correlation analysis showed a significant correlation between SUV and V_D_, (r = 0.85, *p* < 0.01), but no significant relationship between SUV and K_i_ values (r = 0.31, *p* = 0.22). These data indicate a strong association between SUV and V_D_ measurements but no strong relationship between SUV and K_i_ values (Fig. 2).

**Table 2:** Individual V_D_, SUV and K_i_ values in regions identified by abnormally elevated VD, SUV and K_i_ values (see also Fig. 2)

V_D_			SUV			K_i_			
Pt #	High V_D_	High K_i_	High SUV	High V_D_	High K_i_	High SUV	High V_D_	High K_i_	High SUV
1	0.82	0.53	0.64	2.44	2.09	2.81	0.0019	0.0083	0.0043
2	1.27	0.28	0.93	1.65	2.28	3.21	0.0029	0.0113	0.0074
3	1.16	0.47	0.92	1.72	1.44	1.84	0.0013	0.0072	0.0031
4	1.33	0.18	1.08	2.19	1.27	2.23	0.0041	0.0148	0.0078
5	0.82	0.32	0.55	3.28	2.36	3.31	0.0025	0.0119	0.0054

**Fig. 3:**
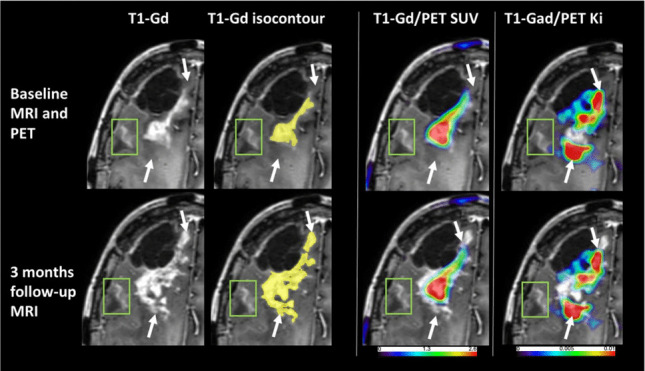
Detection of non-enhancing glioblastoma progression by post-treatment [^18^F]FETrp PET (patient #2). PET was done to evaluate two new contrast-enhancing lesions in the right frontal lobe > 6 months after resection and chemo-radiation. The right lateral frontal contrast-enhancing area (green box) showed no [^18^F]FETrp increases (both SUV and K_i_ below threshold); this area remained stable on subsequent MRI 3 months later, indicating radiation injury (bottom panel). The larger medial mass showed high [^18^F]FETrp SUV mostly confined within the contrast-enhancing volume, while high K_i_-values extended behind (also anterior to) this mass into non-enhancing brain (arrows). Follow-up MRI 3 months later showed expansion of contrast enhancement into these high-K_i_ areas, consistent with tumor progression in this lesion. Corresponding time-activity curves of this patient representing regions with high Ki and high SUV values together with Patlak-plots are provided in the Supplementary Data section

**Fig. 4:**
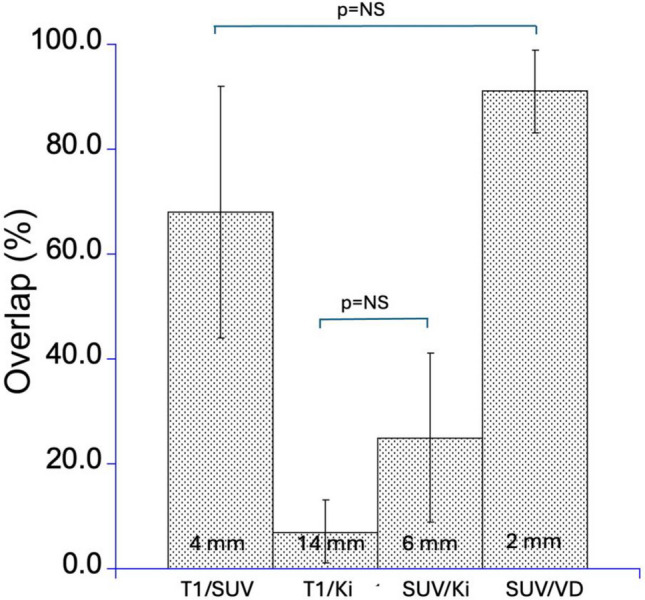
The Figure shows the mean spatial overlap (%) and the mean Euclidean distance (mm, displayed at the bottom of the corresponding bars) between gadolinium-enhanced regions on structural MRI (T1-gad) and PET-derived regions of elevated SUV, V_D_ and K_i_. High spatial concordance is observed between contrast-enhancing MRI abnormalities and regions of increased SUV, characterized by greater regional overlap and shorter inter-regional distances. In contrast, areas defined by elevated K_i_—indicative of enhanced metabolic trapping—demonstrate reduced spatial correspondence with MRI-defined abnormalities as well with SUV, characterized by lower overlap percentages and increased spatial separation. Finally, there is a very high (> 90%) overlap between SUV and V_D_ measures, indicating that V_D_ provides only minimal added information to SUV measures. All comparisons are significant at the 5% level except for those labeled as (*p* = NS)

Spatial relationship among regions presenting with MRI vs. PET abnormalities: Regions demonstrating elevated SUV and K_i_ values generally coincided with the peripheral areas of structural abnormalities identified by T1-gadolinium-enhanced (T1-gad) or FLAIR MR imaging, with the high K_i_ area localized predominantly in non-enhancing brain. Figures 1 and 3 provide a representative illustration of the spatial relationships among the abnormal regions defined by these parameters. Patient #2 also had a second, peripheral contrast-enhancing area that showed low PET values, not exceeding the established thresholds, and was deemed to be radiation injury on subsequent MRIs (Fig. 3). Table 3 shows individual values of overlap as well as the distance between the centroids of abnormally increased V_D_, K_i_ and SUV. Specifically, a substantial overlap of 68 ± 24% (average distance of 4.5 ± 2.2 mm) was observed between contrast-enhancing regions and areas of abnormally elevated SUV. In contrast, the overlap between contrast-enhancing regions and regions of elevated K_i_ values was significantly lower (7 ± 6%, *p* < 0.01), accompanied by a greater average spatial separation of 14.1 ± 11.8 mm, indicating that the bulk of high K_i_ area was localized outside the contrast enhancing mass in adjacent brain tissue. Moreover, the overlap between regions with elevated SUV and K_i_ values was moderate, measured at 24 ± 17% with an average distance of 6.5 ± 4.3 mm (Fig. 4). Notably, a very high spatial correlation was found between regions exhibiting elevated SUV and V_D_ values, showing an overlap of 91 ± 8% and a minimal average distance of 2.1 ± 1.2 mm, indicating strong spatial concordance between these two parameters. Figure 4 also demonstrates that the spatial overlap between regions with elevated K_i_ and those with increased SUV or T1-gadolinium enhancement is low (< 25%), indicating that K_i_ mapping captures distinct pathophysiological information beyond structural abnormalities or tracer accumulation alone.

**Table 3:** Percentage overlap relative to non-overlapping areas (%) among regions with MRI-defined structural abnormalities (contrast-enhancing area defined by T1gad) and PET-derived functional abnormalities characterized by tracer uptake (SUV), the unidirectional uptake rate constant (K_i_) and the apparent volume of distribution (V_D_). Abnormalities were defined as value > 2SD of control brain regions. The Euclidean distance (mm) between regional centroids (centers of gravity) is provided in brackets (see also Fig. 4). Patient #5 had MRI done more than one month of the PET, therefore, the contrast-enhancing area was not compared with the PET volumes

Pt #	T1gad – SUV	T1gad – K_i_	SUV – K_i_	SUV – V_D_
1	87 (2.6)	16 (10.3)	34 (6.7)	98 (1.7)
2	31 (7.3)	0 (33.4)	0 (13.7)	95 (1.2)
3	71 (3.8)	7 (7.2)	17 (5.3)	78 (2.9)
4	84 (2.6)	6 (6.6)	43 (3.9)	94 (1.9)
5	n/a	n/a	33 (3.1)	90 (2.3)

Interestingly, in patients who had MRI follow-up without new treatment after the PET scans (see details in Table 1), these MRIs showed expansion of the contrast-enhancing mass into the area(s) of high K_i_, consistent with tumor progression (seen in patient #2 and #5, see example on Fig. 3). In two other patients with high K_i_ values (#1, #4), subsequent resection included portions of the high K_i_ area and verified tumor presence. In patient #4, with a large, heterogeneous contrast-enhancing frontal lobe mass, separate tissue specimens were obtained from the medial and lateral portions: while the medial portion, showing the highest K_i_ values, showed densely cellular solid tumor on histopathology, the lateral portion of the enhancing mass (with low values on PET), was described as mostly having extensive necrotic areas (with some tumor cell presence), consistent with radiation injury. In one patient (#3, with a small parietal contrast-enhancing lesion), the follow-up MRIs were initially stable, for up to 5 months, showing only minimal change, suggesting that the lesion itself was mostly radiation injury; this patient had the lowest K_i_ values (Table 2). Additional follow-up MRIs then showed expansion of the contrast-enhancing area, indicating late tumor progression.

## Discussion

Our study demonstrates the potential clinical relevance of [^18^F]FETrp parametric image generation for improved assessment of glioblastomas. Specifically, this methodology allows the quantification of both amino acid transport and tumoral metabolic changes that are related to increased tryptophan metabolism linked to the upregulated of the immunosuppressive KYN pathway. In addition to establishing normative cortical values for K_i_, SUV and V_D_ for the [^18^F]FETrp tracer, the main results of our study are as follows: (i) Tumor-associated regions showed significantly elevated K_i_ accompanied by increased V_D_ and SUV values; however, there was a notable spatial mismatch between areas of high K_i_ and those with elevated SUV/V_D_; (ii) Regions with elevated SUV and V_D_ showed a marked overlap with the contrast-enhancing mass on T1-weighted imaging, whereas areas of elevated K_i_ predominantly extended into non-enhancing (often FLAIR-hyperintense) regions, potentially indicating sites of future tumor progression; (iii) The strong spatial correlation between SUV and V_D_ suggests limited incremental value of V_D_ for characterizing tumor tissue beyond what is provided by SUV alone. Overall, our study highlights the clinical potential of the [^18^F]FETrp tracer in combination with parametric image generation using methodological advances in image acquisition/reconstruction that allow this approach to be easily implemented in routine clinical application.

### Added Value of [^18^F]FETrp Map of the Unidirectional Uptake Rate Constant

The ability of [^18^F]FETrp uptake and trapping to reflect not only amino acid transport but also metabolic trapping, linked to the activity of the KYN pathway, sets this tracer apart from other amino acid PET radiotracers [27, 29]. Human gliomas, especially glioblastomas, almost invariably upregulate key enzymes of the KYN pathway, and this plays a central role in glioma-associated immune tolerance [40, 41], which can be reversed by specific KYN pathway enzyme inhibitors [42, 43]. Tryptophan-based molecular imaging holds the promise to evaluate such drug effects *in vivo* [44], and [^18^F]FETrp K_i_ metabolic maps may provide a refined tool to monitor treatment effects in future clinical trials. Our previous studies with [^11^C]AMT PET imaging also demonstrated the ability of this technique to detect non-enhancing glioma-infiltrated brain, even without using metabolic parametric maps [45, 46]. The present study extends this observation to post-treatment glioblastomas and indicates that areas with high K_i_ values can extend well beyond even high SUV areas. Subsequent expansion of contrast enhancement to these high K_i_ regions (as illustrated on Fig. 3) supports the notion that these areas indeed represent glioma-infiltrated brain. Pending further confirmation, these high K_i_ regions could be considered for inclusion in targeted therapy, such as re-resection or reirradiation, to improve survival. It should be noted that lower (below-threshold) K_i_ values within the contrast-enhancing mass may be either due to lack of tumor activity (such as radiation injury) and/or due to a massive break-down of the blood-tumor-barrier leading to rapid tracer leakage that can distort the slope parameter in the Patlak plot and lead to falsely low values. These areas may show high V_D_ values within the contrast-enhancing lesion, with high overlap volumes with the high SUV area, indicating that static SUV and parametric V_D_ volumes largely reflect the same process driven by rapid tracer transport rather than metabolic trapping. Overall, the combination of high SUV (or V_D_) and K_i_ areas may yield the most accurate approximation of the full tumor mass, including both enhancing and non-enhancing portions. Again, this notion requires further studies with image-guided histopathology correlations, which were not available for the current study.

### Non-invasive Parametric Image Generation of [^18^F]FETrp K_i_

In routine clinical practice, static PET imaging primarily relies on visual assessment, which necessitates optimal image quality and sufficient contrast between target and background regions. Semiquantitative SUV measurements are commonly utilized to complement visual interpretation, particularly for clinical monitoring and response assessment. However, SUV-based measurements have inherent methodological constraints that may adversely affect accurate PET/CT interpretation. These limitations are especially relevant in therapeutic response evaluations, where differences in image acquisition times following tracer administration can yield inconsistent results. Additionally, SUV measurements are influenced by various technical and physiological factors, including hardware calibration, imaging protocols, and individual patient characteristics. In contrast, multiparametric images derived from dynamic multi-pass PET scans represent an emerging technique in clinical PET that deserves greater utilization in the clinical arena. Compared to conventional SUV images, parametric images provide additional information that is based on tissue dynamics and not solely based on tracer concentration at one particular point in time. As a result, parametric images allow differentiation between tissues with different kinetic properties not possible with static SUV measures that only reflect the tracer concentration at the end of the PET scan.

Moreover, it is important to highlight the methodological advancement in both PET image acquisition and reconstruction that allows automated *non-invasive* generation of parametric Ki images. The dynamic multi-pass protocol automatically defines an image-derived input function from the center of the LV and uses direct reconstruction from PET raw data to produce brain K_i_ maps [47]. This approach yields parametric images markedly less susceptible to noise compared to images derived via the traditional indirect image-based (post-reconstruction) method [48]. Consequently, the resultant multiparametric images can display good visual quality.

### Limitations

The main limitation of our study is the small number of subjects, due to the currently limited use of the [^18^F]FETrp tracer reserved only for select research patients. However, once the clinical relevance of K_i_ maps for identification of regions of early tumor progression is established, we anticipate increased demand for [^18^F]FETrp imaging in brain tumor patients. Another potential limitation of this approach in clinical routine might be the relatively long scan time of 60 min which may be inconvenient and prone to motion artifact. Since tracer accumulation can plateau around 40 min post-injection [35], a shortened acquisition time (40–50 min) can be considered for future studies once it is validated, consistent with published practice guidelines for dynamic amino acid PET imaging [10]. Nevertheless, the potential benefit of this method in providing valuable clinical information with regard to the location of early tumor progression might offset this logistic hurdle. Future studies could also aim to refine the optimal cutoff threshold to delineate high [^18^F]FETrp uptake and K_i_ areas; in the present study, we used a threshold based on 2SD above the normal values measured in contralateral cortex. Future studies in larger samples can test various thresholds and compare these with outcomes, such as subsequent progression volumes or image-guided biopsy results. Such studies would also benefit from blood radio-metabolite measurements, as unexpected metabolites in humans may require metabolite correction to ensure that the quantification results fully reflect the true tracer behavior. Finally, the quantitative estimates reported in this study may be influenced by the use of an IDIF that has not been fully validated for the [^1^⁸F]FETrp tracer.

## Conclusion

Overall, the main clinical relevance of our study is the establishment of a novel quantitative molecular imaging biomarker that is non-invasively obtained (without the need for invasive blood sampling) and that can be used to generate quantitative parametric maps of tryptophan transport and metabolic activity that allow improved detection of tumor-infiltrated non-enhancing brain tissue to facilitate accurate differentiation between tumor progression and radiation injury post-treatment. Finally, the applied imaging approach may also serve as a valuable tool for evaluating the metabolic impact of pharmacologic interventions targeting the immunosuppressive KYN pathway, with the potential to reverse its detrimental effects and improve clinical outcome.

## Supplementary Information

Below is the link to the electronic supplementary material.

ESM 1 Supplementary Material 1 (DOCX 1.39 MB)

## Data Availability

Data will be available upon request.
