# Supplementary material for: Preliminary Results on the Added Value of Parametric Images Derived from 18F-fluoroethyl-L-tryptophan PET for Posttreatment Glioblastoma Assessment
Source: Mol Imaging Biol. 2025 Dec 22;28(1):116–26. doi: 10.1007/s11307-025-02075-4 (PMC12966211; doi:10.1007/s11307-025-02075-4)
Supplement: Supplementary file 1 — Supplementary Material 1 (DOCX 1.39 MB) [file 11307_2025_2075_MOESM1_ESM.docx]

**Supplementary data section**

**Figure S1** shows two representative image-derived input functions (IDIFs) used for the generation of parametric images. Both curves exhibit a well-defined bolus peak within the first 2 minutes post-injection, followed by a gradual decline to low background blood activity. The close similarity in the temporal profiles of the two IDIFs - with comparable bolus dispersion and consistently low late-time activity - indicates high reproducibility and suggests that there is minimal spillover contamination from adjacent myocardial activity into the left-ventricular blood-pool signal.


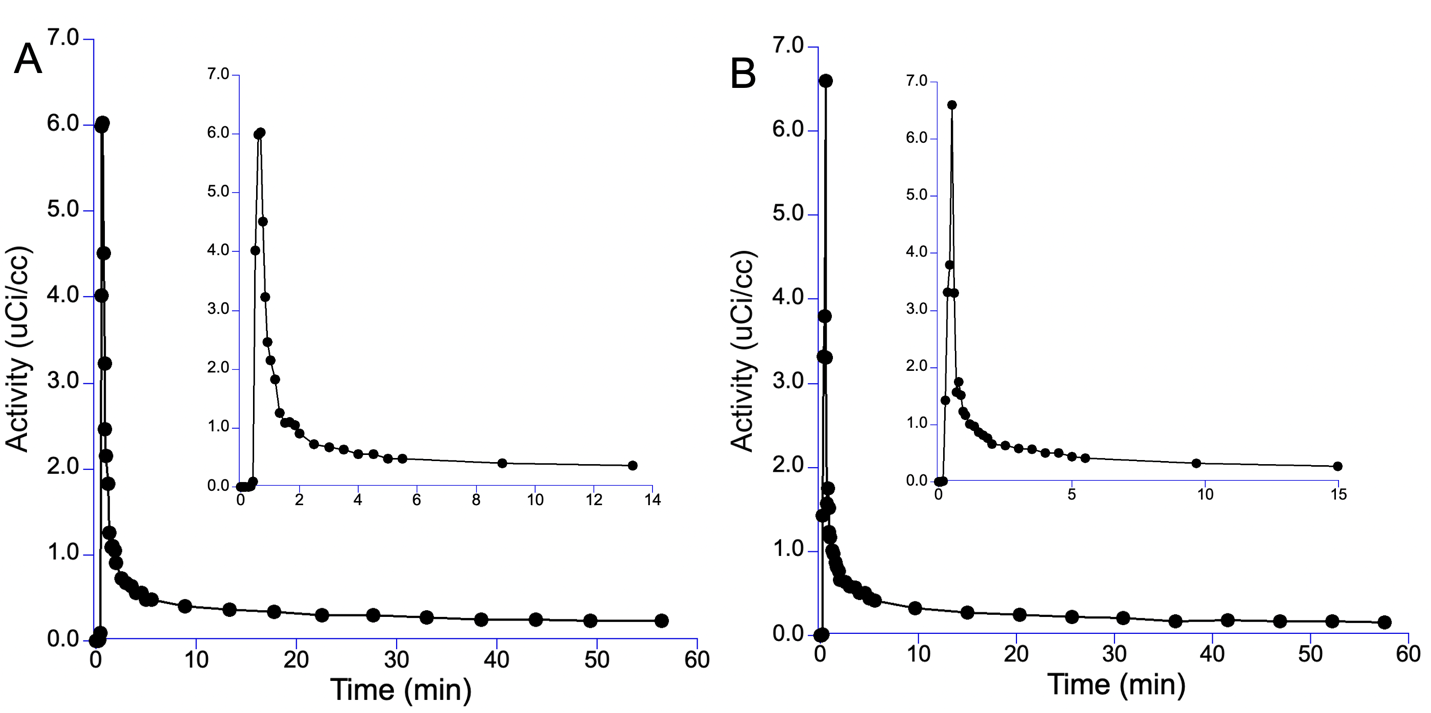


**Figure S1.** Representative image-derived input functions (IDIFs). Panels A and B show two representative IDIFs over the full 0–60 min post-injection (p.i.) interval. Insets display the first 15 minutes to highlight the bolus peak and early blood tracer kinetics. Each IDIF was generated by concatenating the initial high-temporal-resolution dynamic heart acquisition (0–5 min) with the heart-focused portion of the subsequent multi-pass dynamic protocol (11 × 5-min frames). The smooth continuity between the initial dynamic sequence and the later time frames demonstrates the consistency of the blood-pool signal across acquisition stages.

**Figure S2** illustrates [^18^F]FETrp kinetics in normal control tissue and tumor regions from patient #1 (corresponding to Figure 1 in the main manuscript). Panel A displays the time–activity curves (TACs) fitted with a two-tissue compartment model. Non-linear curve fitting was performed using a standard Marquardt-Levenberg least-squares algorithm implemented using the MATLAB software package. Kinetic modeling enables the differentiation between tracer transport into tissue and its subsequent metabolic processing. Transport between the blood pool and tissue is characterized by the unidirectional influx rate constant K_1_ (ml/min) and the efflux rate constant k₂ (1/min), whereas metabolic trapping of tracer in tissue is characterized by the metabolic rate constant (k₃, 1/min). The expression K_1_k_3_/(k_2_+k_3_) represents the unidirectional uptake rate constant Kᵢ which corresponds to the slope parameter in the Patlak graphical analysis. The volume of distribution (V_D_) prior to substantial irreversible binding is given by the expression K_1_/k_2_, which aligns with the intercept in the Patlak plot. Compartmental analysis revealed a markedly higher unidirectional uptake rate constant K_i_ in regions with elevated K_i_ (0.00836) compared with high-SUV regions (0.00491) and control tissue (0.00287). Panel B shows the corresponding Patlak plots, demonstrating that slope estimates (0.00828, 0.00465, and 0.00284) derived from data 30-55 min p.i. closely matches the compartmental model-derived K_i_ values, confirming the validity of the linearization approach.

**
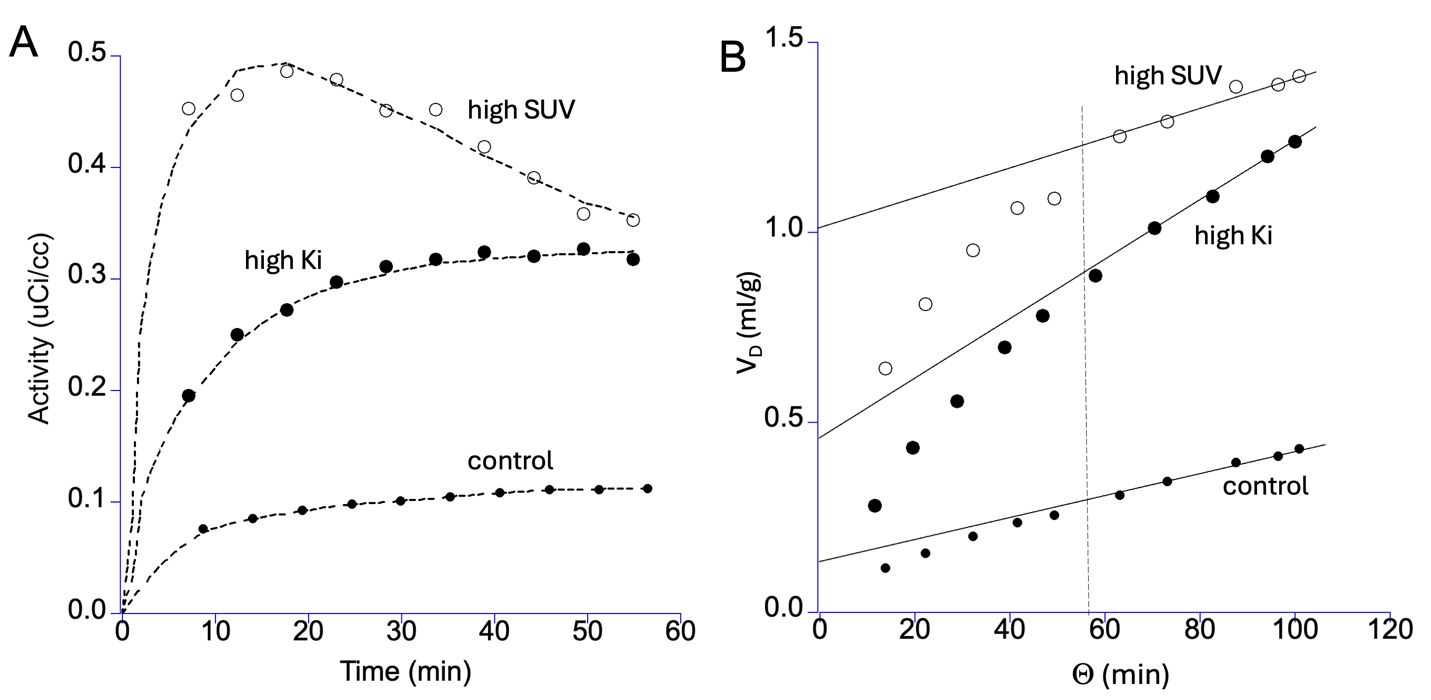
**

**Figure S2.** **(A)** Representative time-activity curves (TACs) derived from ROIs in the contralateral control cortex (control) and ipsilateral areas defined by elevated K_i_ (high K_i_) or elevated SUV (high SUV) determined in patient #1. High SUV regions showed a steep initial rise in tracer uptake followed by pronounced washout beginning ~15 minutes post-injection. Conversely, high K_i_ regions and control tissue displayed a continuously rising profile with sustained tracer retention, reflecting reduced efflux and increased irreversible binding. Accordingly, TACs were fitted using a two-tissue compartment model (broken lines) yielding estimates of the K_i_ macroparameter. **(B)** Corresponding Patlak graphical analysis of control, high K_i_ and high SUV TACs with linear fits to data obtained from 30 to 55min p.i. (indicated by broken line) yielding the slope parameter. The parameter Θ represents the Patlak-transformed blood input function defined as [
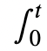
C_b_(τ)dτ]/C_b_(t) and V_D_ is the apparent volume of distribution. The graph demonstrates linearity of data after ~30min p.i.

Similarly, **Figure S3** presents [^18^F]FETrp kinetics in control tissue and tumor regions from patient #2 (corresponding to Figure 2 in the main manuscript). **Panel A** depicts TACs fitted with a two-tissue compartment model, while **Panel B** shows the corresponding Patlak plots. The Patlak-derived slope estimates (high-K_i_ region = 0.00958, high-SUV region = 0.00528, control region = 0.00298), calculated over the 30–55 min post-injection interval, closely matched the K_i_ values obtained from full compartmental modeling (0.00977 for high-K_i_ region, 0.00546 for high-SUV region and 0.00301 for control tissue). This close agreement further supports the validity of the Patlak graphical analysis as a reliable method for quantifying [18F]FETrp kinetics in a clinical setting.

**
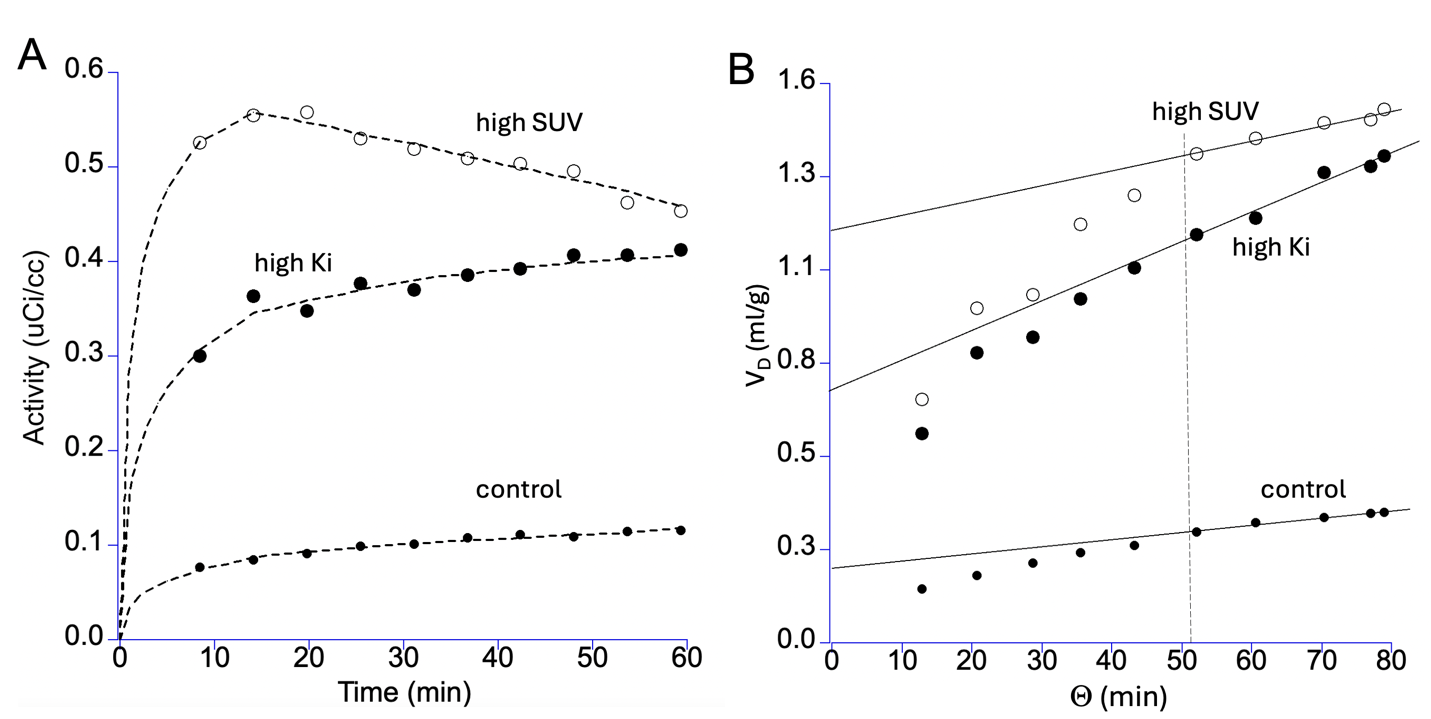
**

**Figure S3.** **(A)** Representative time-activity curves (TACs) derived from ROIs in the contralateral control cortex (control) and ipsilateral areas defined by elevated K_i_ (high K_i_) or elevated SUV (high SUV) determined in patient #2. TACs were fitted using a two-tissue compartment model (broken lines) yielding estimates of the K_i_ macroparameter. **(B)** Corresponding Patlak graphical analysis of control, high K_i_ and high SUV TACs with linear fits to data obtained from 30 to 55min p.i. (indicated by broken line). The parameter Θ represents the Patlak-transformed blood input function defined as [
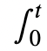
C_b_(τ)dτ]/C_b_(t) and V_D_ is the apparent volume of distribution.
